# A computational algorithm for optimal design of a bioartificial organ scaffold architecture

**DOI:** 10.1371/journal.pcbi.1012079

**Published:** 2024-11-11

**Authors:** Martina Bukač, Sunčica Čanić, Boris Muha, Yifan Wang

**Affiliations:** 1 Department of Applied and Computational Mathematics and Statistics, University of Notre Dame, South Bend, Indiana, United States of America; 2 Department of Mathematics, University of California, Berkeley, California, United States of America; 3 Department of Mathematics, Faculty of Science, University of Zagreb, Zagreb, Croatia; 4 Department of Mathematics and Statistics, Texas Tech University, Lubbock, Texas, United States of America; North Carolina State University, UNITED STATES OF AMERICA

## Abstract

We develop a computational algorithm based on a diffuse interface approach to study the design of bioartificial organ scaffold architectures. These scaffolds, composed of poroelastic hydrogels housing transplanted cells, are linked to the patient’s blood circulation via an anastomosis graft. Before entering the scaffold, the blood flow passes through a filter, and the resulting filtered blood plasma transports oxygen and nutrients to sustain the viability of transplanted cells over the long term. A key issue in maintaining cell viability is the design of ultrafiltrate channels within the hydrogel scaffold to facilitate advection-enhanced oxygen supply ensuring oxygen levels remain above a critical threshold to prevent hypoxia. In this manuscript, we develop a computational algorithm to analyze the plasma flow and oxygen concentration within hydrogels featuring various channel geometries. Our objective is to identify the optimal hydrogel channel architecture that sustains oxygen concentration throughout the scaffold above the critical hypoxic threshold.

The computational algorithm we introduce here employs a diffuse interface approach to solve a multi-physics problem. The corresponding model couples the time-dependent Stokes equations, governing blood plasma flow through the channel network, with the time-dependent Biot equations, characterizing Darcy velocity, pressure, and displacement within the poroelastic hydrogel containing the transplanted cells. Subsequently, the calculated plasma velocity is utilized to determine oxygen concentration within the scaffold using a diffuse interface advection-reaction-diffusion model. Our investigation yields a scaffold architecture featuring a hexagonal network geometry that meets the desired oxygen concentration criteria. Unlike classical sharp interface approaches, the diffuse interface approach we employ is particularly adept at addressing problems with intricate interface geometries, such as those encountered in bioartificial organ scaffold design. This study is significant because recent developments in hydrogel fabrication make it now possible to control hydrogel rheology and utilize computational results to generate optimized scaffold architectures.

## Author summary

Bioartificial organ is an engineered device made of living cells and a biocompatible scaffold that can be implanted or integrated into a human body to replace a natural organ, or to augment a specific organ function. The biocompatible scaffold serves as the structural foundation, embedding cells and growth factors to form substitute tissue. A key challenge in bioartificial organ design is ensuring the long-term survival of transplanted cells. Specifically, a critical challenge is creating a hydrogel architecture with ultrafiltrate channels that act as pathways to deliver oxygen and nutrients to the cells, supporting their sustained viability. In this work, we developed a mathematical and computational framework to investigate optimal hydrogel architecture and oxygen supply to transplanted cells. Our findings show that a hexagonal channel architecture significantly outperforms both the commonly used vertical channels and branching channel designs, providing a substantial increase in oxygen delivery to the cells. In the hexagonal architecture, oxygen concentration remains above the critical threshold for hypoxia throughout the scaffold, which is not achieved with other designs. This is particularly important given recent advances in hydrogel fabrication, allowing for precise control of hydrogel elasticity and rheology, making these results valuable for developing improved scaffold designs.

## 1 Introduction

**Fig 1:**
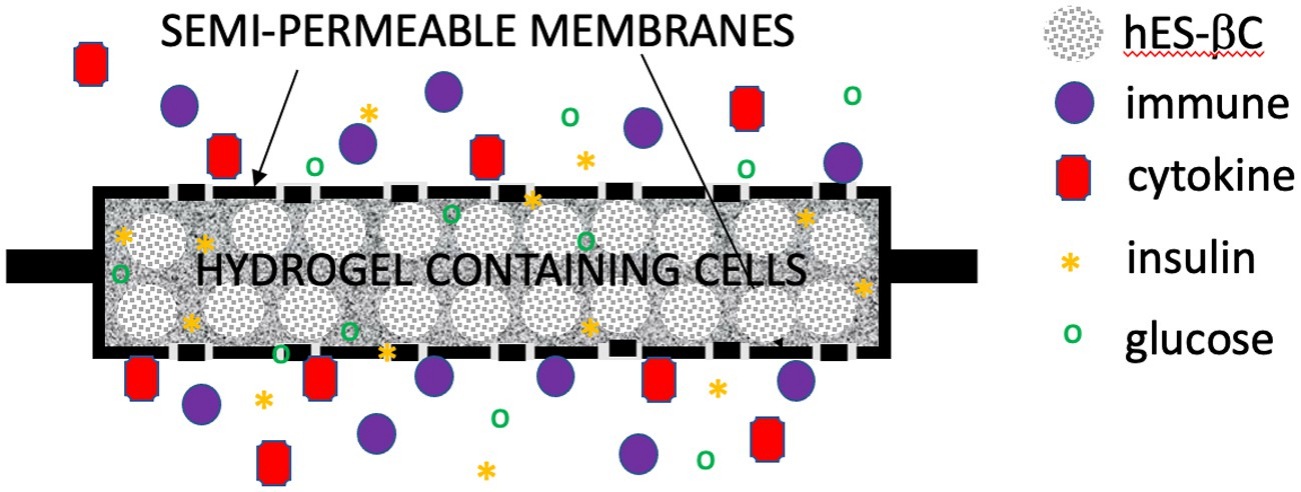
Schematic illustration of immunoprotective encapsulation device containing human embryonic stem cell-derived β clusters (hES-βC).

Bioartificial organ is an engineered device made of living cells and a biocompatible scaffold that can be implanted or integrated into a human body–interfacing with living tissue–to replace a natural organ, or to duplicate or augment a specific organ function [1]. Biocompatible scaffold is a biocompatible base material in which cells and growth factors are embedded to construct a substitute tissue, which can be used in e.g. bioartificial organ design [2]. For example, a biocompatible scaffold used in the bioartificial pancreas design is a biocompatible hydrogel (e.g. agarose gel) which houses transplanted pancreatic islets (conglomerations of pancreatic cells) that contain the insulin-producing *β*-cells. Hydrogels are hydrophilic polymers that are poroelastic, and that have the ability to absorb a large volume of fluid, which makes them particularly suitable materials for biomedical applications. In bioartificial pancreas design presented in [3], the biocompatible hydrogel containing the insulin-producing cells is encapsulated in a nano-pore, semi-permeable membrane (filter), see Fig 1, and the resulting bioartificial organ is connected to the host’s cardiovascular system via an anastomosis graft (a tube) for the advection-enhanced nutrients delivery to the cells, and insulin distribution away from the cells.

A major challenge in the encapsulation-based bioartificial organ design is maintaining the survival of transplanted cells within the bioartifical organ scaffold for an extended period of time. In particular, long-term viability of pancreatic cells is directly affected by the sufficient access to nutrients for survival, of which oxygen is the limiting factor [4].

One of the goals of this manuscript is to develop the mathematical and computational approaches to investigate oxygen supply to the transplanted cells by studying hydrogel architecture and optimal design of ultrafiltrate channels within the encapsulated poroelastic hydrogels for advection-enhanced reaction-diffusion processes of oxygen transport to the cells. This is significant because recent developments in hydrogel fabrication make it now possible to control hydrogel elasticity and hydrogel rheology using, e.g., the approaches employed and reviewed in [2, 5].

In this manuscript we study oxygen-carrying blood plasma flow and oxygen concentration in three different scaffold architectures, whose geometries are inspired by different biological flow-nutrients scenarios. The first geometry consists of vertical ultrafiltrate channels drilled through a hydrogel, which is the simplest, and a standard procedure used in the design of bioartificial pancreas [5–7]. The second geometry consists of the branching channels, inspired by the architecture of the branching vessels in the human body. The third geometry consists of the channels surrounding hexagonal pockets, which we refer to as the hexagonal geometry. This was inspired by the biological (epithelial) tissues in which interstitial fluid flows through a network of irregularly arranged interstices between hexagonally shaped cells, which supports their structural and functional integrity [8]. To work with comparable fluid flow scenarios, the three geometries are generated so that the total fluid channels’ volume (i.e., the area of the 2*D* channels) is the same in all three geometries.

**Fig 2:**
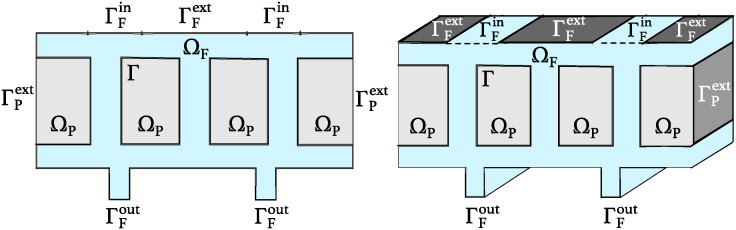
An example of a domain Ω = Ω_*F*_ ∪ Ω_*P*_ demonstrating the two inlets $\Gamma_{F}^{in}$ at the top and the two outlets $\Gamma_{F}^{out}$ at the bottom in 2*D* (left) and 3*D* (right).

We are interested in a scaffold architecture with the geometry that provides concentration of oxygen that is as uniform throughout the scaffold as possible and above a minimum concentration below which hypoxia occurs. For this purpose we introduce two sets of models, one for the fluid flow and one for oxygen concentration in the scaffold. The scaffold is modeled as a 2*D* domain containing a network of channels separating the regions of poroelastic medium describing a hydrogel. See Fig 2 below. In the last part of the manuscript we introduce a 3*D* computational model to show that for the “optimal” geometry, the 2*D* simulations capture the main features of flow and oxygen concetration. The flow of blood plasma through the network of channels is modeled by the time dependent Stokes equations for an incompressible, viscous fluid. The channel flow is coupled to the filtrating flow in the poroelastic hydrogel, which is modeled by the Biot equations [9]. Two sets of coupling conditions at the interface between the channel flow and hydrogel poroelastic medium are employed: the kinematic and dynamic coupling conditions. The kinematic coupling conditions describe continuity of normal (vertical to the interface) velocity and a slip in the tangential component of the velocity, known as the Beaver-Joseph-Saffman condition. The dynamic coupling conditions state the balance of forces at the interface. Linear coupling is considered, i.e., the coupling conditions are evaluated at a reference interface. Subsequently, the resulting fluid velocity is used as an input data in an advection-reaction-diffusion model for oxygen concentration within the scaffold.

To solve the coupled problems computationally, we implemented a diffuse interface approach [10–12]. The diffuse interface approach has several advantages over the classical sharp interface approach. In the diffuse interface approach, the mesh nodes do not have to be aligned with the interface, whose location is now captured using a phase-field function. Instead, the problem unknowns are defined on the entire domain, and the mesh remains fixed. This is particularly useful in cases where the interface between the two regions is difficult to determine exactly (e.g. “fuzzy” boundaries between regions obtained from medical images), or when the geometry of the interface is complex, leading to inaccurate approximations of the boundary integrals. Furthermore, advantages of this approach are apparent in problems with moving geometries and in the development of geometric optimization algorithms for optimal channel architecture design, which we plan to do next. In these cases a diffuse interface approach avoids the creation of complicated meshes following optimizing scaffold geometries, which would be the case in the classical sharp interface approaches. In this work we develop a diffuse interface approach to solve both the fluid and oxygen problems. In both cases, a finite element method is used for spatial discretization, and a backward Euler method for time stepping. We perform a verification of our numerical diffuse interface approach by comparing the solutions of the diffuse interface problem with the already validated solutions of a sharp interface approach, showing excellent agreement. Additionally, to justify the computationally less expensive 2*D* simulations, for the optimal geometry we perform the full 3*D* simulations and show that the 2*D* simulations we used to find the optimal geometry, capture all the important features of plasma flow and oxygen concentration in the corresponding 3*D* scaffold.

The diffuse interface approach is then used to investigate flow, pressure, and oxygen concentration in the three main geometries, discussed above. As mentioned earlier, the diffuse interface approach developed here is particularly suitable from the computational standpoint to study a number of different interface geometries. We find that the ultrafiltrate channel network with the hexagonal geometry provides the best solution in terms of oxygen concentration distribution and magnitude. Oxygen concentration in the hexagonal case is most uniformly distributed throughout the entire scaffold, and the values of concentration are all well above the critical value of oxygen below which hypoxia occurs. This is not the case for the other two geometries considered here (vertical channels and branching channels). We then further investigate the reasons why the hexagonal geometry performs best, and conclude that the relative angle of the channels with respect to the flow direction is one of the main contributing factors to higher Darcy velocity within the poroelastic hydrogel, and consequently the higher concentration values in between the ultrafiltrate channels. The results obtained in this manuscript can be used in optimal scaffold design by implementing hydrogel manufacturing techniques recently developed in [2, 5].

This manuscript is organized as follows. In Section 2 we introduce the model equations in strong/differential and weak/integral formulations. In Section 3 the diffuse interface method is discussed, and the fluid and oxygen problems are presented in the weak (integral) diffuse interface formulations. Numerical discretization based on a finite element approach is also discussed in this section. The computational setting, which includes the computational geometries described by the phase-field function, and parameter identification, is presented in Section 4. In this section we also show the verification of the numerical solver by comparing the diffuse interface solution with a monolithic solver utilizing a sharp-interface approach. Numerical results are presented in Section 5. In Section 6, we present 3*D* simulations of the optimal geometry demonstrating strong agreement with the 2*D* simulations. Conclusions are presented in Section 7.

## 2 Methods I: The mathematical model

To model the entire scaffold, we introduce a bounded domain $\Omega \subset R^{d}$, with *d* = 2, 3, consisting of a fluid region, Ω_*F*_, corresponding to the blood or blood plasma flow region bringing oxygen and nutrients to the transplanted cells, and a poroelastic solid region, Ω_*P*_, corresponding to the poroelastic medium such as a biocompatible hydrogel, which hosts the transplanted cells. The entire bioartificial organ scaffold is then comprised of the two regions so that $\bar{\Omega }={\bar{\Omega }}_{F}\cup {\bar{\Omega }}_{P}$ and Ω_*F*_ ∩ Ω_*P*_ = ∅ (see Fig 2). The fluid flowing through the channels comprising the region Ω_*F*_ enters the poroelastic medium Ω_*P*_ across the interface Γ, which is the interface between Ω_*F*_ and Ω_*P*_, defined by $\Gamma ={\bar{\Omega }}_{F}\cap {\bar{\Omega }}_{P}$.

Fig 2 shows a sketch of a 2*D* and a 3*D* domain in which the fluid domain Ω_*F*_ consists of two horizontal channels corresponding to the “inlet” and “outlet” channels, namely, they contain the inlet boundary $\Gamma_{F}^{\text{in}}$ and the outlet $\Gamma_{F}^{\text{out}}$, respectively. The two horizontal channels are connected via three vertical channels describing the simplest scaffold architecture which has been used in several research studies on bioartificial organ design [5–7]. The 3*D* geometry is obtained by extruding the 2*D* geometry. The vertical ultrafiltrate channels are drilled to enhance advection-dominated oxygen and nutrients supply to the cells residing in Ω_*P*_. This is just one, and the simplest example of the scaffold architecture that we will consider in this manuscript.

To model the interaction between the flow of blood plasma in Ω_*F*_ and the filtration of blood plasma through Ω_*P*_, we will use a fluid-poroelastic structure interaction model, which we present next. We assume that the fluid-poroelastic structure interaction problem is linear, and that the displacement is small enough so that the domain remains fixed.

### 2.1 Fluid-poroelastic structure interaction

We assume that Ω_*F*_ contains a viscous, incompressible, Newtonian fluid, such as blood plasma, whose dynamics can be described by the Stokes equations:

$$\begin{array}{cc} \rho_{F}\partial_{t}v=\nabla \cdot \sigma_{F}\; & \text{in}\;\Omega_{F}\times \left(0,T\right), \end{array}$$ (1a)

$$\begin{array}{cc} \nabla \cdot v=0\; & \text{in}\;\Omega_{F}\times \left(0,T\right), \end{array}$$ (1b)

where ***v*** stands for the fluid velocity, *ρ*_*F*_ is density, and ***σ***_*F*_ is the Cauchy stress tensor defined by:

$$\begin{array}{c} \sigma_{F}\left(v,p_{F}\right)=-p_{F}I+2\mu_{F}D\left(v\right), \end{array}$$ (2)

where *p*_*F*_ represents the fluid pressure, *μ*_*F*_ is the fluid viscosity, and ***D***(***v***) = (∇***v*** + ∇***v***^*τ*^)/2 is the strain rate tensor. Namely, Eq (2) is a constitutive law describing a Newtonian fluid such as blood plasma. Velocity ***v*** will be used in the advection-diffusion-reaction problem to convect the oxygen concentration in Ω_*F*_.

The poroelastic medium, such as agarose gel used in the design of a bioartificial pancreas, see [4], can be described by the well-known Biot model, which has been used to describe hydrogels in [13]. The Biot model consists of the following equations:

$$\begin{array}{c} \rho_{S}\partial_{tt}\eta +\gamma_{E}\eta =\nabla \cdot \sigma_{P}\;\text{in}\;\Omega_{P}\times \left(0,T\right), \end{array}$$ (3a)

$$\begin{array}{c} -\kappa \nabla p_{P}=q\;\text{in}\;\Omega_{P}\times \left(0,T\right), \end{array}$$ (3b)

$$\begin{array}{c} \partial_{t}\left(c_{0}p_{P}+\alpha \nabla \cdot \eta \right)+\nabla \cdot q=0\;\text{in}\;\Omega_{P}\times \left(0,T\right). \end{array}$$ (3c)

Eq (3a) describes the elastodynamics of the elastic skeleton, i.e., the solid phase in the Biot model, and is given in terms of the displacement of the elastic skeleton, denoted by ***η***, from its reference configuration Ω_*P*_. Constant *ρ*_*S*_ denotes density of the poroelastic matrix, and *γ*_*E*_ is a spring coefficient. For the simulations performed in 2*D*, adding the term *γ*_*E*_***η*** accounts for the three-dimensional elastic energy effects, and helps to keep the regions that are not connected from drifting away. More precisely, this term helps to prevent spurious numerical solutions where sections of Ω_*P*_ might be excessively pulled in the direction of vertical fluid flow. Such unrealistic large vertical displacements do not occur in 3*D* models due to the presence of an elastic restoring force in the third spatial dimension. In 3*D* simulations this term is not needed, so in that case we set *γ*_*E*_ = 0.

The total Cauchy stress tensor ***σ***_*P*_ for the poroelastic region is defined by

$$\sigma_{P}\left(\eta ,p_{P}\right)=\sigma_{E}\left(\eta \right)-\alpha p_{P}I,$$

where *α* is the Biot-Willis parameter accounting for the coupling strength between the fluid and solid phases and ***σ***_*E*_ denotes the elastic stress tensor, described by the Saint Venant-Kirchhoff constitutive model as:

$$\sigma_{E}\left(\eta \right)=2\mu_{E}D\left(\eta \right)+\lambda_{E}\text{tr}\left(D\left(\eta \right)\right)I,$$

where λ_*E*_ and *μ*_*E*_ are Lamé parameters. Eq (3b) is the Darcy law, where *p*_*P*_ is the fluid pore pressure, ***q*** is the Darcy velocity, and *κ* is the permeability. Eq (3c) is the storage equation for the fluid mass conservation in the pores of the matrix. It describes the coupled behavior of fluid flow and solid deformation in a porous medium. In this context, the quantity *c*_0_*p*_*P*_ + *α*∇ ⋅ ***η*** represents the total fluid content, where *c*_0_ is a constant related to the fluid’s compressibility. The mass conservation equation incorporates the effects of both fluid and solid compressibility to ensure that the total mass is conserved, considering how the fluid pressure changes impact the solid matrix and vice versa. The Darcy velocity ***q*** will be used in the advection-diffusion-reaction problem to convect the oxygen concentration in Ω_*P*_.

#### Coupling conditions

To couple the fluid flow model with the Biot poroelastic medium model we use a set of four coupling conditions: two kinematic coupling conditions and two dynamic coupling conditions, which are evaluated at the location Γ of the interface between the two models. To state the coupling conditions introduce ***n***_*F*_ to denote the outward unit normal vector to ∂Ω_*F*_. At the interface Γ the following coupling conditions hold:

$$\begin{array}{c} \left(v-\partial_{t}\eta \right)\cdot n_{F}=q\cdot n_{F}\;\text{on}\;\Gamma \times \left(0,T\right), \end{array}$$ (4a)

$$\begin{array}{c} \gamma {\left(v-\partial_{t}\eta \right)}_{\tau }=-{\left(\sigma_{F}n_{F}\right)}_{\tau }\;\text{on}\;\Gamma \times \left(0,T\right), \end{array}$$ (4b)

$$\begin{array}{c} \sigma_{F}n_{F}\cdot n_{F}=-p_{P}\;\text{on}\;\Gamma \times \left(0,T\right), \end{array}$$ (4c)

$$\begin{array}{c} \sigma_{F}n_{F}=\sigma_{P}n_{F}\;\text{on}\;\Gamma \times \left(0,T\right), \end{array}$$ (4d)

where for a vector ***v***, we define a projection onto the local tangent plane on Γ as:

$${\left(v\right)}_{\tau }≔v-\left(v\cdot n_{f}\right)n_{f}.$$

The first two conditions provide information about kinematic quantities such as velocities (kinematic coupling conditions), and the second two conditions provide information about coupling of stresses/forces (dynamic coupling conditions). More precisely, Eq (4a) describes the fluid mass conservation across the interface, i.e., the normal components of the free fluid velocity ***v*** relative to the velocity of motion of the poroelastic matrix ∂_*t*_***η*** is equal to the Darcy velocity ***q*** across the interface. Eq (4b) describes the Beavers-Joseph-Saffman condition (4b) with slip rate *γ* > 0, namely, the tangential component of the free fluid velocity slips at the interface with the slip rate proportional to the fluid shear stress (***σ***_*F*_***n***_*F*_)_***τ***_. Eqs (4c) and (4d) describe the continuity of pressures and total stresses at the interface.

#### Boundary and initial conditions

Problem (1a)–(4d) is supplemented with boundary and initial conditions. In our notation, we represent the exterior boundary as Γ^*ext*^ = ∂Ω. The exterior boundary is divided into three distinct parts: the fluid inflow boundary, the fluid outflow boundary, and the impenetrable part of the boundary, $\Gamma^{ext}=\Gamma_{F}^{in}\cup \Gamma_{F}^{out}\cup \Gamma_{F}^{ext}$. For the fluid problem, we impose the following boundary conditions:

$$v=v_{in}\;\;\text{on}\;\Gamma_{F}^{in}\times \left(0,T\right),$$

$$v=0\;\;\text{on}\;\Gamma_{F}^{ext}\times \left(0,T\right),$$

$$\sigma_{F}n_{F}=0\;\;\text{on}\;\Gamma_{F}^{out}\times \left(0,T\right),$$

where ***v***_*in*_ is a prescribed velocity, specified in Section 4.

For the poroelastic structure, we impose no flow and zero displacement at the external boundaries:

$$\begin{array}{cc} q\cdot n_{P}=0\; & \;\text{on}\;\Gamma_{P}^{ext}\times \left(0,T\right), \end{array}$$ (5)

$$\begin{array}{cc} \eta =0\; & \;\text{on}\;\Gamma_{P}^{ext}\times \left(0,T\right). \end{array}$$ (6)

Initially, we assume that the fluid is at rest and that the deformable poroelastic structure is in its reference configuration. Thus, we have:

$$\begin{array}{c} v=0,\;\eta =0,\;\partial_{t}\eta =0,\;p_{P}=0\;\text{at}\;t=0. \end{array}$$ (7)

#### Weak formulation

To define the weak formulation of problem (1a)–(7) we introduce the following function spaces. Given an open set *S*, we consider the usual Sobolev spaces *H*^*k*^(*S*), with *k* ≥ 0, and introduce the following function spaces:

$$\begin{array}{cc} & V_{F}\left(\Omega_{F}\right)={\left\{v\in \left(H^{1}\Omega_{F}\right)\right)}^{d}\;:\;v=0\;\text{on}\;\Gamma_{F}^{in}\cup \Gamma_{F}^{ext}\},\\ & V_{F,D}\left(\Omega_{F}\right)={\left\{v\in \left(H^{1}\Omega_{F}\right)\right)}^{d}\;:\;v=0\;\text{on}\;\Gamma_{F}^{ext},\;\;v=v_{in}\;\text{on}\;\Gamma_{F}^{in}\},\\ & V_{E}\left(\Omega_{P}\right)=\left\{\eta \in H^{1}\left(\Omega_{P}\right)\;:\;\eta =0\;\text{on}\;\Gamma^{ext}\right\}. \end{array}$$

Here $V_{F}\left(\Omega_{F}\right)$ corresponds to the test space for the fluid velocity, $V_{F,D}\left(\Omega_{F}\right)$ is associated with the solution space for the fluid velocity, and $V_{E}\left(\Omega_{P}\right)$ is associated with the function space for the poroelastic matrix displacement. The spaces *H*^1^(Ω_*P*_) and *L*^2^(Ω_*F*_) are associated with the solutions spaces for the fluid pressures in Ω_*p*_ and Ω_*F*_ respectively.

We say that $\left(v,p_{P},\eta ,p_{F}\right)\in L^{2}\left(0,T;V_{F,D}\left(\Omega_{F}\right)\right)\times H^{1}\left(\Omega_{P}\right)\times L^{\infty }\left(0,T;V_{E}\left(\Omega_{P}\right)\right)\times H^{-1}\left(0,T;L^{2}\left(\Omega_{F}\right)\right)$ is a weak solution if for every $\left(w,\psi_{P},\zeta ,\psi_{F}\right)\in V_{F}\left(\Omega_{F}\right)\times H^{1}\left(\Omega_{P}\right)\times V_{E}\left(\Omega_{P}\right)\times L^{2}\left(\Omega_{F}\right)$, the following equality is satisfied in $D^{\prime }\left(0,T\right)$:

$$\begin{array}{c} \frac{d}{dt}(\int_{\Omega_{F}}\rho_{F}v\cdot w\;dV+\int_{\Omega_{P}}\rho_{S}\partial_{t}\eta \cdot \zeta \;dV+c_{0}\int_{\Omega_{P}}p_{P}\psi_{P}\;dV)\\ +2\mu_{F}\int_{\Omega_{F}}D\left(v\right):D\left(w\right)\;dV-\int_{\Omega_{F}}\left(\nabla \cdot w\right)p_{F}\;dV+\int_{\Omega_{F}}\left(\nabla \cdot v\right)\psi_{F}\;dV\\ +\int_{\Omega_{P}}\kappa \nabla p_{P}\cdot \nabla \psi_{P}\;dV+\int_{\Omega_{P}}\gamma_{E}\eta \cdot \zeta \;dV+2\mu_{E}\int_{\Omega_{P}}D\left(\eta \right):D\left(\zeta \right)\;dV\\ +\lambda_{E}\int_{\Omega_{P}}\left(\nabla \cdot \eta \right)\left(\nabla \cdot \zeta \right)\;dV-\alpha \int_{\Omega_{P}}\left(\nabla \cdot \zeta \right)p_{P}\;dV+\alpha \int_{\Omega_{P}}\left(\nabla \cdot \xi \right)\psi_{P}\;dV\\ +\gamma \int_{\Gamma }{\left(v-\partial_{t}\eta \right)}_{\tau }{\left(w-\zeta \right)}_{\tau }\;dA-\int_{\Gamma }\psi_{P}\left(v-\partial_{t}\eta \right)\cdot n_{F}\;dA\\ +\int_{\Gamma }p_{P}\left(w-\zeta \right)\cdot n_{F}\;dA=0. \end{array}$$ (8)

Here, $D^{\prime }\left(0,T\right)$ denotes the space of distributions on (0, *T*), which is the dual space of the space of test functions $D\left(0,T\right)=C_{c}^{\infty }\left(0,T\right)$. We note that the weak formulation is obtained using the primal formulation for the Biot problem, i.e., Eqs (3b) and (3c) have been combined so that Eq (3c) is written only in terms of *p*_*P*_ and ***η***. The Darcy velocity can be computed by postprocessing using (3b).

Once the fluid velocity ***v*** and Darcy velocity ***q*** are computed from the fluid-poroelastic structure interaction problem specified above, we can use this velocity information to formulate an advection-reaction-diffusion problem describing oxygen transport in the bioartificial organ consisting of ultrafiltrate channels Ω_*F*_ and the poroelastic medium Ω_*P*_ containing the cells.

### 2.2 Advection-reaction-diffusion

To model the transport of oxygen, we use an advection-reaction-diffusion model describing oxygen transport in the fluid domain Ω_*F*_ and in the poroelastic medium Ω_*P*_. Oxygen transport in the human vascular system and tissues has been studied by many authors [14–18], and we adopt the approach from [15] to study oxygen transport in the scaffold:

$$\begin{array}{cc} \partial_{t}c+u\cdot \nabla c-\nabla \cdot \left(D\nabla c\right)=R_{max}\frac{c}{c+c_{MM}}H\left(c,c_{CR}\right)\; & \text{in}\;\Omega \times (0,T_{t}), \end{array}$$ (9)

where *c* is concentration of oxygen, and *D* is a diffusion coefficient equal to *D*_*F*_ in Ω_*F*_ and to *D*_*P*_ in Ω_*P*_. The velocity ***u*** is equal to ***v*** in Ω_*F*_, and to ***q*** in Ω_*P*_. The final time is *T*_*t*_, which we note is different from the final time *T* used in the fluid-poroelastic structure interaction problem. The reaction term on the right-hand side is active in Ω_*P*_ only and it accounts for the consumption of oxygen in the scaffold. In particular, this reaction term depends on the maximum oxygen consumption rate, *R*_*max*_ < 0, the Michaelis-Menten constant, *c*_*MM*_, corresponding to the oxygen concentration when the consumption rate drops to 50% of its maximum [15], and the function $H(c,c_{CR})$, defined by

$$H\left(c,c_{CR}\right)=\{\begin{array}{cc} 0, & x\in \Omega_{F}\\ 0, & x\in \Omega_{P}\;\text{and}\;c\leq c_{CR}\\ 1, & x\in \Omega_{P}\;\text{and}\;c>c_{CR}, \end{array}$$

accounts for the regions of the tissue/poroelastic matrix where the oxygen concentration falls below a critical concentration *c*_*CR*_ below which necrosis is assumed to occur [15, 19]. The values of the parameters are all specified in [15, 19].

#### Boundary and initial conditions

Eq (9) is supplemented with the following boundary conditions:

$$\begin{array}{cc} c=c_{in}\; & \;\text{on}\;\Gamma_{F}^{in}\times \left(0,T_{t}\right), \end{array}$$ (10a)

$$\begin{array}{cc} c=0\; & \;\text{on}\;\Gamma_{F}^{ext}\times \left(0,T_{t}\right), \end{array}$$ (10b)

$$\begin{array}{cc} -D\nabla c\cdot n=0\; & \;\text{on}\;\Gamma_{F}^{out}\times \left(0,T_{t}\right), \end{array}$$ (10c)

where ***c***_*in*_ is a given quantity, specified in Section 4. Thus, these boundary conditions say that we have a prescribed oxygen concentration at the inlet, zero oxygen concentration at the top boundary of the scaffold between the inlet regions, and zero diffusive oxygen flux at the outlet.

Initially, the concentration is set to zero:

$$\begin{array}{c} c=0\;\text{at}\;t=0. \end{array}$$ (11)

#### Weak formulation

To write the weak formulation of problem (9)–(11) we introduce the following function spaces:

$$\begin{array}{c} M=\left\{c\in H^{1}\left(\Omega \right)\cap L^{\infty }\left(\Omega \right)\;:\;c=0\;\text{on}\;\Gamma_{F}^{in}\cup \Gamma_{F}^{ext}\right\},\\ M_{D}=\left\{c\in H^{1}\left(\Omega \right)\cap L^{\infty }\left(\Omega \right)\;:\;c=0\;\text{on}\;\Gamma_{F}^{ext},\;\;c=c_{in}\;\text{on}\;\Gamma_{F}^{in}\right\}. \end{array}$$

Here M is associated with the test space for oxygen concentration and $M_{D}$ is associated with the solution space for *c*.

We say that $c\in L^{2}\left(0,T;M_{D}\right)$ is a weak solution if *c* ≥ 0 and if for every s∈M, the following equality is satisfied in $D^{\prime }\left(0,T_{t}\right)$:

$$\begin{array}{c} \frac{d}{dt}\int_{\Omega }cs\;dV+\int_{\Omega }\left(u\cdot \nabla c\right)s\;dV+\int_{\Omega }D\nabla c\cdot \nabla s\;dV=\int_{\Omega }R_{max}\frac{c}{c+c_{MM}}H\left(c\right)s\;dV. \end{array}$$ (12)

We have now specified two problems: a fluid-poroelastic structure interaction problem and an advection-reaction-diffusion problem, that we would like to solve for the fluid velocity and poroelastic structure displacement, and for oxygen concentration. The plan for this manuscript is to investigate three different scaffold architectures, motivated by biological structures, and numerically test which one provides the scaffold architecture with oxygen concentration that is closest to the uniform distribution of oxygen and is above the known minimal value *c*_*opt*_ for which uninhibited insulin production by the *β*-cells is guaranteed [20]. See Section 5. For this purpose we developed two numerical methods, one for the fluid-poroelastic structure interaction problem, and one for the advection-reaction-diffusion problem, which we describe next.

## 3 Methods II: Numerical method

**Fig 3:**
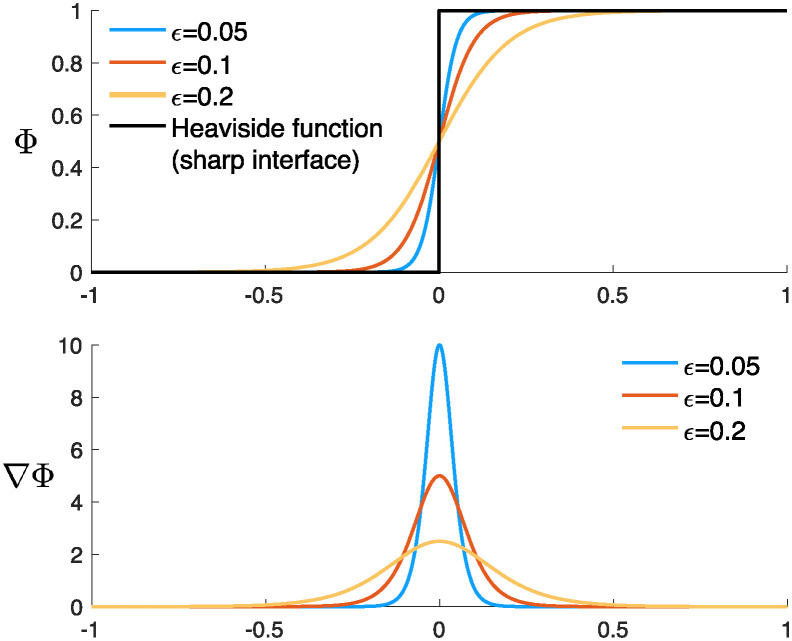
Graphical representation of the diffuse interface approach in one dimension. Top: The phase-field function Φ. Bottom: The gradient of Φ used to approximate the location of the interface.

To solve the fluid-poroelastic structure interaction and transport problems numerically, we use the diffuse interface method [10, 11]. Let *χ* denote the Heaviside function which equals one in Ω_*F*_ and zero in Ω_*P*_. Let a phase-field function Φ: Ω → [0, 1] be a regularization of the Heaviside function such that Φ ≈ 1 in Ω_*F*_, Φ ≈ 0 in Ω_*P*_, and Φ smoothly transitions between these two values on a “diffuse” layer of width *ϵ* (see Fig 3). We suppose that *dA* ≈ |∇Φ|*dV* and $n\approx -\frac{\nabla \Phi }{\left|\nabla \Phi \right|}$. Using this notation, for functions *F* and *f* defined on Ω, we can write:

$$\begin{array}{c} \int_{\Omega_{F}}F\;dV=\int_{\Omega }F\chi \;dV\approx \int_{\Omega }F\Phi \;dV,\\ \int_{\Omega_{P}}F\;dV=\int_{\Omega }F\left(1-\chi \right)\;dV\approx \int_{\Omega }F\left(1-\Phi \right)\;dV,\\ \int_{\Gamma }f\;dA=\int_{\Omega }f\delta_{\Gamma }\;dV\approx \int_{\Omega }f\left|\nabla \Phi \right|\;dV, \end{array}$$

where *δ*_Γ_ is a Dirac distribution at the interface Γ.

Using the approximations above, and $n\approx -\frac{\nabla \Phi }{\left|\nabla \Phi \right|}$, we can write the diffuse interface formulation of problem (8) as follows: Find $\left(v,p_{P},\eta ,p_{F}\right)\in L^{2}\left(0,T;V_{F,D}\left(\Omega \right)\times H^{1}\left(\Omega \right)\right)\times L^{\infty }\left(0,T;V_{E}\left(\Omega \right)\right)\times H^{-1}\left(0,T;L^{2}\left(\Omega \right)\right)$ such that for every $\left(w,\psi_{P},\zeta ,\psi_{F}\right)\in V_{F}\left(\Omega \right)\times H^{1}\left(\Omega \right)\times V_{E}\left(\Omega \right)\times L^{2}\left(\Omega \right)$, the following equality holds:

$$\begin{array}{cc} & \frac{d}{dt}(\int_{\Omega }\rho_{F}v\cdot w\Phi \;dV+\int_{\Omega }\rho_{S}\partial_{t}\eta \cdot \zeta \left(1-\Phi \right)\;dV+c_{0}\int_{\Omega }p_{P}\psi_{P}\left(1-\Phi \right)\;dV)\\ & +2\mu_{F}\int_{\Omega }D\left(v\right):D\left(w\right)\Phi \;dV-\int_{\Omega }\left(\nabla \cdot w\right)p_{F}\Phi \;dV+\int_{\Omega }\left(\nabla \cdot v\right)\psi_{F}\Phi \;dV\\ & +\int_{\Omega }\kappa \nabla p_{P}\cdot \nabla \psi_{P}\left(1-\Phi \right)\;dV+\int_{\Omega }\gamma_{E}\eta \cdot \zeta \left(1-\Phi \right)\;dV\\ & +2\mu_{E}\int_{\Omega }D\left(\eta \right):D\left(\zeta \right)\left(1-\Phi \right)\;dV+\lambda_{E}\int_{\Omega }\left(\nabla \cdot \eta \right)\left(\nabla \cdot \zeta \right)\left(1-\Phi \right)\;dV\\ & -\alpha \int_{\Omega }\left(\nabla \cdot \zeta \right)p_{P}\left(1-\Phi \right)\;dV+\alpha \int_{\Omega }\left(\nabla \cdot \xi \right)\psi_{P}\left(1-\Phi \right)\;dV\\ & +\gamma \int_{\Omega }{\left(v-\partial_{t}\eta \right)}_{\tilde{\tau }}{\left(w-\zeta \right)}_{\tilde{\tau }}\left|\nabla \Phi \right|\;dV+\int_{\Omega }\psi_{P}\left(v-\partial_{t}\eta \right)\cdot \nabla \Phi \;dV\\ & -\int_{\Omega }p_{P}\left(w-\zeta \right)\cdot \nabla \Phi \;dV=0, \end{array}$$ (13)

where we used the $\tilde{\tau }$ subscript to denote the “approximate” tangential component of a vector function at the diffused interface, defined, for a vector ***v***, as follows:

$${\left(v\right)}_{\tilde{\tau }}≔v-(v\cdot \nabla \Phi )\frac{\nabla \Phi }{{\left|\nabla \Phi \right|}^{2}}.$$

Tangent vectors that form a basis with ∇Φ can also be obtained directly from the phase-field function using the algorithm described in [12, 21].

We note that in order to write (13), the variables on each subdomain have to be extended onto the entire domain Ω. This procedure introduces singularities. For example, when the fluid velocity, which is defined on Ω_*F*_, is extended into the whole domain Ω, the corresponding integrand will be multiplied by Φ. Since Φ is zero in a large part of Ω_*P*_, the resulting linear system will have zero rows whenever this occurs, which will lead to a singular matrix. Therefore, we use the following regularization of Φ:

$$\begin{array}{c} \Phi \approx (1-2\beta )\Phi +\beta , \end{array}$$ (14)

where *β* is a small positive number. Therefore, Φ ≥ *β* and 1 − Φ ≥ *β*. This regularization of Φ was used in the definition of problem (13). A phase-field method for the related Stokes-Darcy problems have been analyzed by the authors in [22] where existence of a weak solution was proved, and its convergence to the sharp interface solution was obtained.

Because the concentration Eq (12) is already defined in Ω, we do not require regularization. In that case, *β* = 0. However, we use Φ to define the global velocity and diffusion:

$$\begin{array}{c} u=v\Phi +q\left(1-\Phi \right),\;\text{and}\;D=D_{F}\Phi +D_{P}\left(1-\Phi \right). \end{array}$$

### Numerical discretization

Problems (13) and (12) are discretized in time using the Backward Euler method, and in space using a finite element method. Since the transport problem requires the fluid velocity, but the fluid-poroelastic structure interaction problem does not require any information from the transport problem, we first solve the fluid-poroelastic structure interaction problem until the final time *T* is reached. Then, we solve the transport problem. Let N∈N be the number of time steps for the fluid problem, *T* the final time, and Δ*t* = *T*/*N* the time step. We define the discrete times *t*^*n*^ = *n*Δ*t*, for *n* = 0, …, *N*. We also denote by *h* a discretization parameter associated with the triangulation $T_{h}\left(\Omega \right)$ of Ω. For each *h*, we choose finite dimensional subspaces $V_{F}^{h}\subset V_{F}\left(\Omega \right),V_{F,D}^{h}\subset V_{F,D}\left(\Omega \right),V_{E}^{h}\subset V_{E}\left(\Omega \right),X_{F}^{h}\subset L^{2}\left(\Omega \right),X_{P}^{h}\subset H^{1}\left(\Omega \right),M^{h}\subset M$ and $M_{D}^{h}\subset M_{D}$ over the triangulation $T_{h}\left(\Omega \right)$. We use MINI elements [23] to approximate the fluid velocity and pressure, $P_{1}$ elements to approximate the Darcy pressure and the displacement, and $P_{2}$ elements to approximate the concentration.

The fully discrete fluid-poroelastic structure interaction problem is given as follows: given $(v_{h}^{0},p_{P,h}^{0},\eta_{h}^{0},p_{F,h}^{0}),$ for *n* = 0, …, *N* − 1, find $\left(v_{h}^{n+1},p_{P,h}^{n+1},\eta_{h}^{n+1},p_{F,h}^{n+1}\right)\in V_{F,D}^{h}\times X_{P}^{h}\times V_{E}^{h}\times X_{F}^{h}$ such that for every $\left(w_{h},\psi_{P,h},\zeta_{h},\psi_{F,h}\right)\in V_{F}^{h}\times X_{P}^{h}\times V_{E}^{h}\times X_{F}^{h}$, the following equality holds:

$$\begin{array}{cc} & \int_{\Omega }\rho_{F}\frac{v_{h}^{n+1}-v_{h}^{n}}{\Delta t}\cdot w_{h}\Phi \;dV+\int_{\Omega }\rho_{S}\frac{\eta_{h}^{n+1}-2\eta_{h}^{n}+\eta_{h}^{n-1}}{\Delta t^{2}}\cdot \zeta_{h}\left(1-\Phi \right)\;dV\\ & +c_{0}\int_{\Omega }\frac{p_{P,h}^{n+1}-p_{P,h}^{n}}{\Delta t}\psi_{P,h}\left(1-\Phi \right)\;dV+2\mu_{F}\int_{\Omega }D{\left(v_{h}\right)}^{n+1}:D\left(w_{h}\right)\Phi \;dV\\ & -\int_{\Omega }\left(\nabla \cdot w_{h}\right)p_{F,h}^{n+1}\Phi \;dV+\int_{\Omega }\left(\nabla \cdot v_{h}^{n+1}\right)\psi_{F,h}\Phi \;dV+\int_{\Omega }\kappa \nabla p_{P,h}^{n+1}\cdot \nabla \psi_{P,h}\left(1-\Phi \right)\;dV\\ & +\gamma_{E}\int_{\Omega }\eta_{h}^{n+1}\cdot \zeta_{h}\left(1-\Phi \right)\;dV+2\mu_{E}\int_{\Omega }D\left(\eta_{h}^{n+1}\right):D\left(\zeta_{h}\right)\left(1-\Phi \right)\;dV\\ & +\lambda_{E}\int_{\Omega }\left(\nabla \cdot \eta_{h}^{n+1}\right)\left(\nabla \cdot \zeta_{h}\right)\left(1-\Phi \right)\;dV-\alpha \int_{\Omega }\left(\nabla \cdot \zeta_{h}\right)p_{P}^{n+1}\left(1-\Phi \right)\;dV\\ & +\alpha \int_{\Omega }\left(\nabla \cdot \xi^{n+1}\right)\psi_{P}\left(1-\Phi \right)\;dV+\gamma \int_{\Omega }(v_{h}^{n+1}-\frac{\eta_{h}^{n+1}-\eta_{h}^{n}}{\Delta t}{)}_{\tilde{\tau }}{\left(w_{h}-\zeta_{h}\right)}_{\tilde{\tau }}\left|\nabla \Phi \right|\;dV\\ & +\int_{\Omega }\psi_{P,h}(v_{h}^{n+1}-\frac{\eta_{h}^{n+1}-\eta_{h}^{n}}{\Delta t})\cdot \nabla \Phi \;dV-\int_{\Omega }p_{P,h}^{n+1}\left(w_{h}-\zeta_{h}\right)\cdot \nabla \Phi \;dV=0. \end{array}$$ (15)

To solve the transport problem, we let $N_{t}\in N$ be the number of time steps, and Δ*t*_*t*_ = *T*_*t*_/*N*_*t*_ the time step. The fully discrete transport problem reads as follows: for *n* = 0, …, *N*_*t*_ − 1, find $c_{h}^{n+1}\in M_{D}^{h}$ such that for every $s_{h}\in M^{h}$, the following equality holds:

$$\begin{array}{cc} \int_{\Omega }\frac{c_{h}^{n+1}-c_{h}^{n}}{\Delta t_{t}}s_{h}\;dV & +\int_{\Omega }u_{h}^{n+1}\cdot \nabla c_{h}^{n+1}s_{h}\;dV+\int_{\Omega }D\nabla c_{h}^{n+1}\cdot \nabla s_{h}\;dV\\ & =\int_{\Omega }R_{max}\frac{c_{h}^{n}}{c_{h}^{n}+c_{MM}}H\left(c_{h}^{n}\right)s_{h}\;dV. \end{array}$$ (16)

We note that according to (11), initially we have $c_{h}^{0}=0$.

## 4 Computational setting, parameters identification and verification

**Fig 4:**
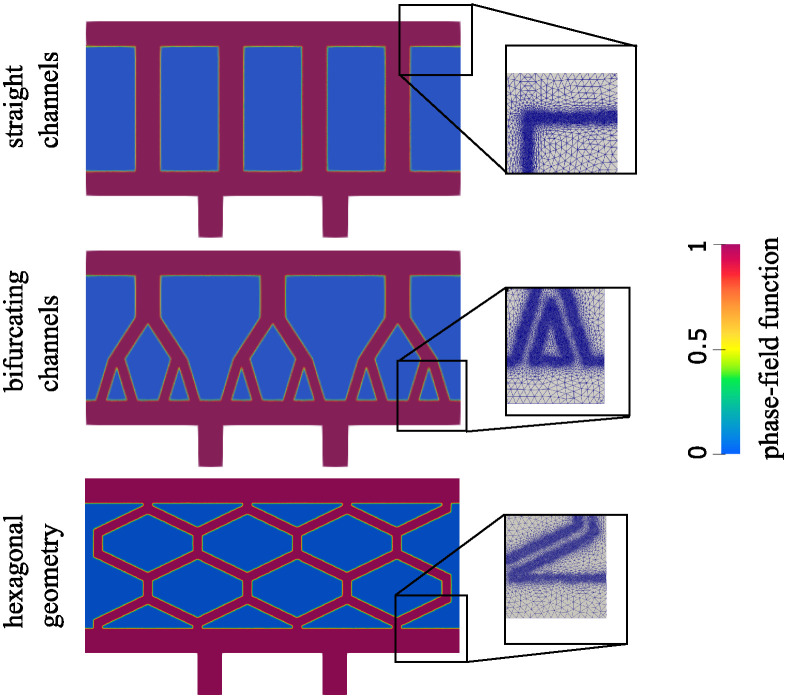
The phase-field function for three different network configurations considered in this work. The zoom-in inserts show the computational mesh. The mesh is refined in the areas with large |∇Φ|, which approximate the interface.

In this work we investigate the fluid velocity, pressure and oxygen concentration in the three geometries/scaffold architectures shown in Fig 4. The first geometry consists of vertical ultrafiltrate channels drilled through a hydrogel, which is the simplest, and a standard procedure used in the design of bioartificial pancreas [3]. The second geometry consists of the branching channels, see the middle panel in Fig 4. This was inspired by the architecture of the branching vessels in the human body. The third geometry consists of the channels surrounding hexagonal pockets, shown at the bottom panel in Fig 4. This geometry was inspired by the biological (epithelial) tissues in which interstitial fluid flows through a network of irregularly arranged interstices between hexagonally shaped cells, which supports their structural and functional integrity [24]. To work with comparable fluid flow scenarios, the three geometries shown in Fig 4 were generated so that the total fluid channels’ area is the same in all three geometries. For both 2*D* and 3*D* simulations, mesh independence tests have been performed. The solutions obtained on the meshes we used in this work do not significantly change if even finer meshes are used.

### 4.1 Computational settings

The size of the computational domain is 0.9 × 0.42 cm, corresponding to the entire scaffold Ω. Two outlet channels are added on the bottom (see Fig 2) to simulate the actual outlet channels shown in Fig 1. Each outlet channel has a height of 0.1 cm and length of 0.06 cm. In each of the three different geometries/architectures of the scaffold Ω, there is a top and a bottom horizontal channel of width 0.06 cm. The same width of 0.6 cm is used for the vertical channels in the first, top configuration. The size of the channels in other configurations is chosen so that the total channel area equals the channel area of the first configuration. In other words, the channels occupy the same area in all three configurations. To computationally capture different geometries/architectures of thin channels distributed within Ω we use a phase-field function. The phase-field function for each of the three geometries is shown in Fig 4. Here, red color corresponds to the value of the phase-field function Φ equal to one, and blue corresponds to Φ = 0. The computational mesh is refined around the interface between the red and blue regions where the gradient of the phase-field function |∇Φ| is large. This is done as follows. We first set Φ = 1 in the region defined by the channels and zero elsewhere. Then, we adapt the mesh and redefine Φ on the finer mesh. To adapt the mesh, we use the *adaptmesh* function in FreeFem++, where we use |∇Φ| as a function which indicates the areas where the mesh should be refined. After that, we solve the following Allen-Cahn problem to allow a smooth transition between 1 and 0:

$$\begin{array}{c} \partial_{t}\Phi -D_{\Phi }\Delta \Phi +\partial_{\Phi }F\left(\Phi \right)=0\;\text{in}\;\Omega \times \left(0,T_{\Phi }\right),\\ \nabla \Phi \cdot n=0\;\text{on}\;\partial \Omega \times (0,T_{\Phi }), \end{array}$$

where *D*_Φ_ = 0.01, *T*_Φ_ = 2 ⋅ 10^−2^, and $F\left(\Phi \right)=2\Phi^{2}{\left(\Phi -1\right)}^{2}-\frac{1}{8}$ is the standard form of a double-well potential [25]. Finally, the procedure of adapting the mesh, redefining Φ and solving the Allen-Cahn problem is repeated one more time. The resulting computational mesh and Φ at time *T*_Φ_ are shown in the zoom-in inserts in Fig 4 (right). We note that this is done only initially since we assume that the fluid-poroelastic structure problem is linear and that the domain is fixed.

### 4.2 Parameter identification

The fluid in Ω_*F*_ represents the filtered blood plasma, which enters the bioartificial organ Ω from an artery via an anastomosis graft, not shown in Fig 4. In encapsulated organs, the blood from the anastomosis graft is filtered through semipermeable nano-pore membranes, and the filtered blood plasma enters a gasket from which the flow of plasma filtrates through the biocompatible hydrogel Ω_*P*_ toward the cells. The horizontal channels represent the gasket containing the blood plasma, and $\Gamma_{F}^{\text{in}}$ corresponds to the location of the semipermeable membranes through which blood plasma enters the top gasket. At $\Gamma_{F}^{\text{in}}$ we prescribe the inlet velocity, which is taken to be ***v***_*in*_ = 3.5 cm/s in 2*D* and ***v***_*in*_ = 5.25 cm/s in 3*D*, so that the flowrate at the inlet is the same in the 2*D* and the 3*D* case. This value was obtained from the three-dimensional numerical simulations of flow through an entire bioartificial pancreas, studied in [9]. The numerical simulations in [9] used the experimentally derived “Darcy-like” relationship between the flow *Q* and pressure gradient Δ*p* through nanoporous membranes reported in [26]: $Q=\frac{w^{3}nl}{12\mu h}\Delta p$, where *Q* is the volumetric flow rate, *w* is the pore width, *l* is the pore length, *h* is the membrane thickness, *n* is the number of pores per (unit) membrane, *μ* is the viscosity, and Δ*p* is the transmembrane pressure. Here, the micro-scale parameters *w*, *l*, *h*, and *n* determine porosity. Therefore, porosity of the membrane influences the calculation of the inlet fluid velocity. The value of 3.5 cm/s agreed well with experiments performed in Dr. Roy’s lab [26]. The parameters used in the blood plasma simulations are standard: the dynamic viscosity is set to *μ*_*F*_ = 0.04 dyn s/cm^2^ and density is *ρ*_*F*_ = 1 g/cm^3^.

The parameters for the poroelastic structure describing the hydrogen scaffold can be obtained from [5, 9]. We took the poroelastic matrix density to be *ρ*_*S*_ = 1.2 g/cm^3^, and the Young’s modulus of 2.5 ⋅ 10^5^ dyne/cm^2^. The Poisson’s ratio is set to 0.49 and the spring constant is *γ*_*E*_ = 10^6^ dyne/cm^4^ in 2*D* and 0 in 3*D*. The spring constant is chosen so that the magnitude of the displacement is of the same order as the one obtained in 3*D*. The pressure storage coefficient is taken as 10^−7^ cm^2^/dyne, and the permeability is *κ* = 10^−5^ cm^3^ s/g. The slip constant is *γ* = 10^3^ g/(cm^2^ s). We set the Biot-Willis parameter *α* to 1. See [9] for the Biot parameters.

Oxygen transport in the human vascular system and tissues has been studied by many authors [14–18, 20]. We adopt the approach from [14] to study oxygen transport in the gasket with oxygen diffusion coefficient given by *D*_*F*_ = 3 ⋅ 10^−5^ cm^2^/s [14]. In the agarose gel, the oxygen diffusion coefficient used in our simulations is *D*_*P*_ = 1.3 ⋅ 10^−5^ cm^2^/s, which is the value that was estimated in rat pancreatic islets and reported in [20, 27]. The maximum oxygen consumption rate is *R*_*max*_ = −3.4 ⋅ 10^−8^ mol/(cm^3^ s), the Michaelis-Menten constant is *c*_*MM*_ = 10^−9^ mol/cm^3^ and the critical oxygen concentration is *c*_*CR*_ = 10^−10^ mol/cm^3^, all obtained from [15]. The concentration of oxygen at the fluid inlet is *c*_*in*_ = 2 ⋅ 10^−7^ mol/cm^3^ [28].

We first perform simulations of the fluid-poroelastic structure interaction problem using Δ*t* = 10^−3^ and *T* = 1 s, which is when a steady state is reached. Using the velocities at the final time of the simulation, we then solve the advection-reaction-diffusion problem with Δ*t*_*t*_ = 5 ⋅ 10^−2^ and *T*_*t*_ = 200 s. All computations are performed within the platform of the finite element software FreeFem++ [29].

To trust the simulations obtained using our diffuse interface method, we validate our computational solver by comparing the results of the diffuse interface method with the results obtained using the “classical” sharp interface approach, on a simpler geometry, as we discuss next. Recall that the main reason for not using the sharp interface approach is the difficulty in generating new scaffold geometries for each new test case. This will be particularly important in our next research phase in which a geometric optimization mathematical model and computational solver will be developed to study optimal design of channels’ distribution in bioartificial organ scaffolds for advection-enhanced oxygen and nutrients supply to the transplanted cells.

### 4.3 Numerical method verification

**Fig 5:**
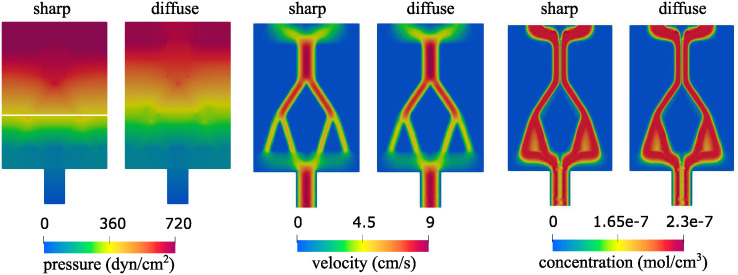
A steady-state solution for the Stokes and Darcy pressure (left) and velocity magnitude (middle) obtained using the sharp interface model and the diffuse interface model. The right two panels shows concentration obtained at *T*_*t*_ = 200. A plot of each of these variables over the line indicated in the leftmost panel is shown in Fig 6.

**Fig 6:**
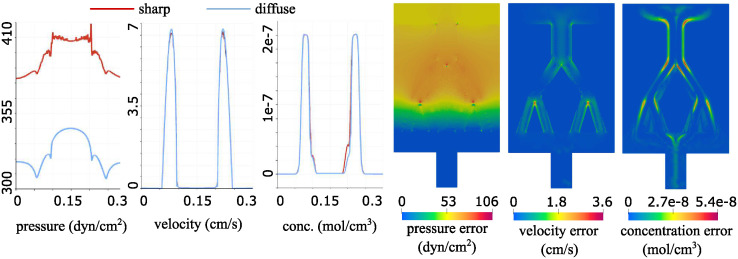
The left three panels show the pressure, velocity and concentration, respectively, obtained using the sharp and the diffuse interface method, plotted over the line indicated in the leftmost panel in Fig 5. The right three panels show errors for the pressure, velocity and concentration, respectively, obtained at steady state.

To validate our diffuse interface solver, we focus on a specific problem characterized by a domain geometry comprising a main channel branching into two, each of which further bifurcates into additional two channels. See Fig 5. We solve the fluid-poroelastic structure interaction problem and the advection-reaction-diffusion problem using a sharp interface approach. The sharp interface solver is based on a classical, monolithic, fluid-poroelastic structure interaction approach. The solver was developed and validated in [30, 31]. In particular, the Backward Euler method is used to discretize the problem in time, and a finite element method is used for spatial discretization. The same finite element spaces are used for the diffuse and sharp interface methods. The results of the sharp interface solver are then compared to the results obtained using our diffuse interface solver discussed in this manuscript. All the parameter settings are the same as the ones described in Section 4. Since the permeability is small, a finer mesh close to the interface is required for both sharp and diffuse interface solvers. We use the same mesh in both cases, consisting of 40,239 points and 80,326 elements. A comparison of the Stokes and Darcy pressure (left), velocity magnitude (middle), and concentration (right) is shown in Fig 5. To better visualize the differences between the solutions, in the left three panels in Fig 6, we show the 1D plot of each of these variables over the line indicated in the leftmost panel in Fig 5. Finally, the right three panels of Fig 6 show the error plots for the pressure, velocity and concentration, respectively. All the results shown here are obtained at the final time. We observe some larger differences in the pressure, and a good agreement for the velocity and concentration. We find the approximations of the pressure to be particularly sensitive in channel flow, especially where the channels are narrow, since in the diffuse interface approach the width of channels slightly differs from the one used in the sharp interface approach due to the diffuse approximation of the interface. However, the main goal of this work is related to the concentration approximation, where we obtain a good match between the two methods.

Encouraged by these results, we use the diffuse interface method described above to study the flow and concentration of oxygen in the geometries shown in Fig 4. This is presented next.

## 5 Results I: Numerical results

**Fig 7:**
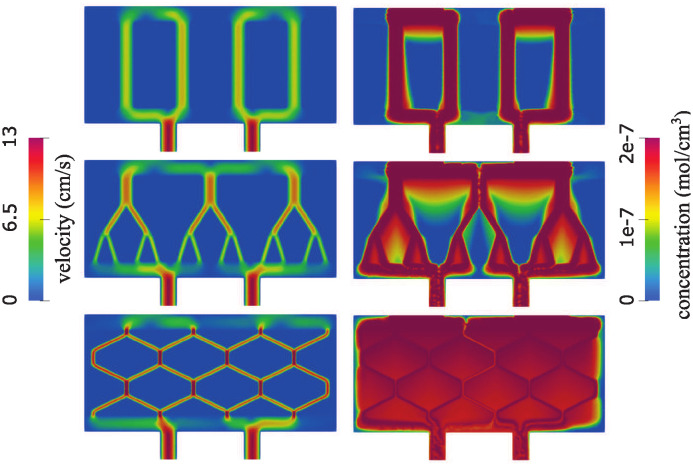
Total velocity magnitude (left) and concentration (right) in a network consisting of straight channels (top), bifurcating channels (middle) and a hexagonal geometry (bottom).

**Fig 8:**
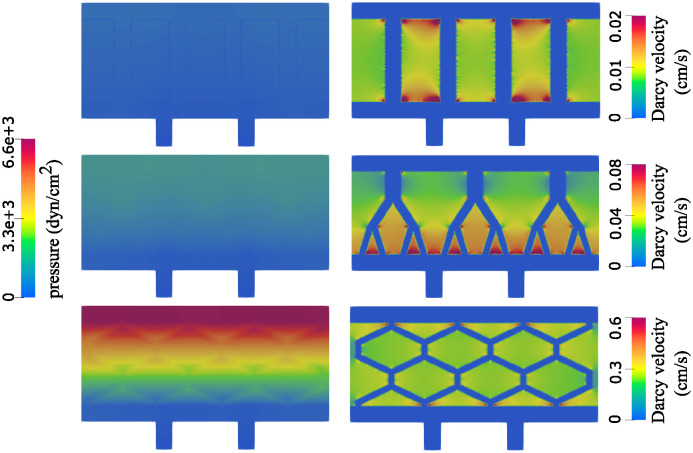
Pressure (left) and the Darcy velocity (right) in a network consisting of straight channels (top), bifurcating channels (middle) and a hexagonal geometry (bottom).

As mentioned earlier, our goal is to study blood plasma flow and oxygen concentration in three different scaffold architectures, as described in Section 4. We are interested in a scaffold architecture that provides concentration of oxygen that is as uniform throughout the scaffold as possible and above the minimum concentration for uninhibited maximal insulin production *c*_*opt*_ = 5 ⋅ 10^−8^ mol/cm^3^, given in [20]. To do this, we use the diffuse interface method to simulate fluid-poroelastic structure interaction providing advection velocity of blood plasma carrying oxygen, and we use the advection-reaction-diffusion solver to calculate oxygen concentration in the entire scaffold, utilizing the advection velocity from the fluid-poroelastic structure interaction simuations. Both models are described in Section 2. The results of the simulations are shown in Figs 7 and 8 below. More precisely, in Fig 7 we plot the total fluid velocity, defined as

u=vΦ+q(1-Φ),

and oxygen concentration for the three different scenarios.

We observe in Fig 7 that in the case of vertically drilled channels, most of the flow is through the vertical channels. The pressure and the Darcy velocity are shown in Fig 8. There are large regions in between the channels where oxygen concentration is below the critical value *c*_*opt*_ = 5 ⋅ 10^−8^ mol/cm^3^ [20], indicating regions of impaired insulin production.

In the case of bifurcating channel network, shown in the middle panels on Figs 7 and 8, we see higher velocity values in the top two generations of branching channels (parent and daughter channels), and larger regions of higher Darcy velocity, shown in light green and yellow, in between the branching vessels. Consequently, we observe larger regions of higher oxygen concentration in between the channels than in the case of vertical channels, see Fig 7 right, middle panel. However, there are still large regions in between the branching trees that have low levels of oxygen concentration, shown in blue, where insulin production is inhibited.

Finally, in the case of the hexagonal geometry, shown in the bottom panels of Figs 7 and 8, the channels have the smallest radius and the fluid velocity is the largest. Darcy velocity in this case is nontrivial in the entire region corresponding to the poroelastic hydrogel. As a result, the oxygen concentration throughout the hydrogel region is high and remains close to the uniform concentration above the critical value *c*_*opt*_. This ensures an adequate oxygen supply to the cells in the entire hydrogel, maintaining levels of oxygen concentration above the critical threshold necessary for normal insulin production.

As a result, oxygen concentration is high in the entire hydrogel region, and it is very close to the uniform concentration above the critical value *c*_*opt*_, as desired, providing oxygen supply to the cells in the entire hydrogel that is above the critical value above which normal insulin production takes place.

**Fig 9:**
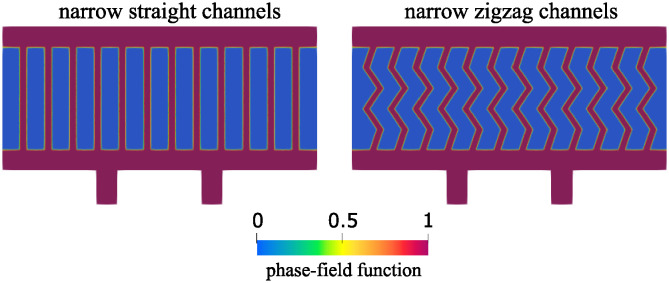
Geometries consisting of narrow straight channels (left) and narrow zigzag channels (right).

**Two additional geometries**. We consider two additional geometries to gain a better understanding of why the hexagonal geometry is associated with improved oxygen concentration levels. Since hexagonal geometry architecture described above has narrower channels that are at an angle with respect to the pressure drop-driven dominant vertical flow, we separate the influence of the two geometric factors and consider the following two geometries: one consisting of narrow vertical channels with a small radius (the width of each channel is equal to one third of the width of vertical channels used above, shown in Fig 4), and the other consisting of the zig-zag (angular) channels with the radius determined by the constraint that the total channel area is the same as in the vertical channel case. See Fig 9.

**Fig 10:**
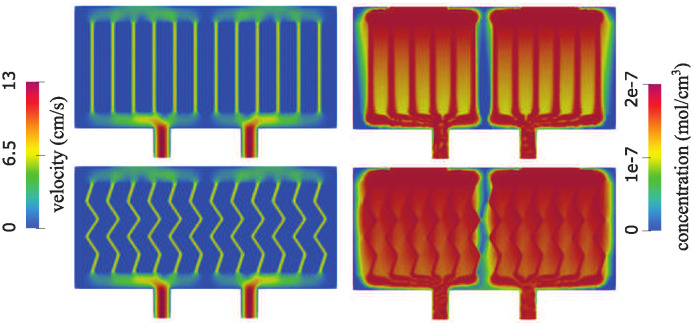
Total velocity magnitude (left) and concentration (right) in a network consisting of narrow straight channels (top) and narrow zigzag channels (bottom).

The flow velocity and concentration obtained in the new geometries are shown in Fig 10. In both geometries, the peak velocity in the channels is equal to 13 cm/s. However, significant differences can be seen in concentration. We note that having narrow channels improves the transport through the poroelastic medium compared to having fewer wider channels (see top-right panel in Fig 7). However, having a network with channels at an angle with respect to dominant flow leads to increased oxygen levels overall, especially at places where the flow changes direction. An explanation for this observation is the fact that flow through porous interfaces is largest when the angle between flow direction and the interface is large.

**Fig 11:**
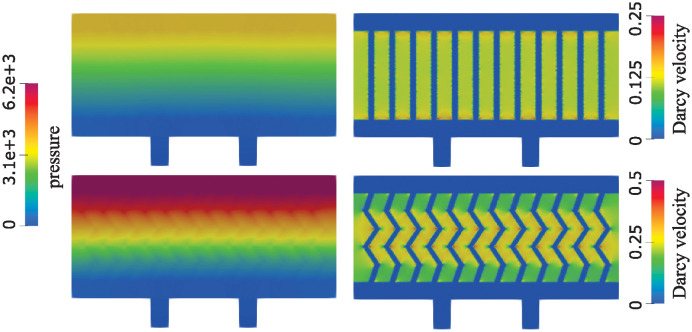
Pressure (left) and the Darcy velocity (right) in a network consisting of narrow straight channels (top) and narrow zigzag channels (bottom).

This is evident in the right panel of Fig 11 where Darcy velocity is shown. The peak Darcy velocity is twice as large when zigzag channels are used compared with the straight channels. To improve the visualization of the peak velocity area, we show the flow in two geometries on different scales. When straight channels are used, the flow is largest closest to the top and bottom of the channels. In zigzag channels, the flow is largest in the middle of the domain. This is an interesting observation that significantly improves oxygen supply to the cells located in the middle of the proelastic hydrogel. We also show the total pressure in the two geometries in Fig 11. Our results show a significantly larger internal scaffold pressure in case of the zigzag network, which is associated with increased Darcy flow through the hydrogel.

**Uninhibited maximal insulin production regions**. Finally, we further quantify the behavior of the three scaffold architectures shown in Fig 4 by considering the performance of the three geometries in terms of the uninhibited maximal insulin production by the transplanted *β*-cells [20]. Namely, motivated by the results in [20] we use a threshold for uninhibited maximal insulin production by the transplanted islets of *c*_*opt*_ = 5 ⋅ 10^−8^ mol/cm^3^ to identify the regions within tissue in which the islet function is compromised. More precisely, we investigate the regions within each scaffold in which the oxygen concentration is higher than the threshold for uninhibited maximal insulin production, *c*_*opt*_, and compare the areas of those regions that support islet function. This area is computed only in the poroelastic domain using the following expression:

$$\int_{\Omega }\chi_{opt}\left(1-\Phi \right)\;dV,\;\text{where}\;\;\chi_{opt}=\{\begin{array}{cc} 1 & \text{if}\;c\geq c_{opt}\\ 0 & \text{otherwise}. \end{array}$$

**Fig 12:**
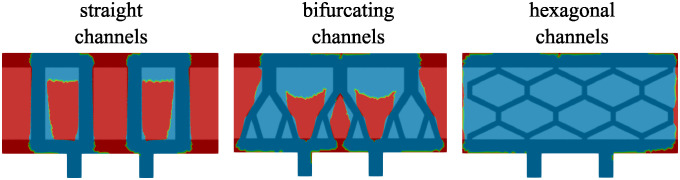
Function *χ*_*opt*_ superimposed with the phase-field function indicating the channel geometry. Regions in red show areas where the insulin production is inhabited, while regions in blue show areas of uninhibited insulin production.

The regions are visualized in Fig 12. Blue color shows the regions of uninhibited insulin production, while the insulin production is compromised in the regions shown in red. The results are quantified in Table 1.

**Table 1:** The area where the concentration is larger than the oxygen threshold of uninhibited maximal insulin production for all the geometries considered in this manuscript.

Geometry	Total area (cm^2^)	Relative area
straight network	0.026	13.23%
bifurcating network	0.102	51.74%
hexagonal geometry	0.192	97.13%
narrow straight network	0.172	86.94%
narrow zigzag network	0.187	94.24%

As expected, the hydrogel geometry with the smallest area of oxygen levels above *c*_*opt*_ is obtained for the network consisting of straight channels (13% of total area), followed by the bifurcating channels, which have the uninhibited maximal insulin production area equal to 51.74% of the total area. Finally, the most efficient insulin production is observed in the hexagonal geometry, where more than 97% of the poroelastic region is above the uninhibited maximal insulin production threshold *c*_*opt*_. We note that in the hexagonal geometry, the largest red regions are close to the lateral sides of the domain. This is because we impose a no flux condition on $\Gamma_{P}^{ext}$ and no flow on $\Gamma_{F}^{ext}$, which explains the boundary layers.

## 6 Results II: Three-dimensional simulations

**Fig 13:**
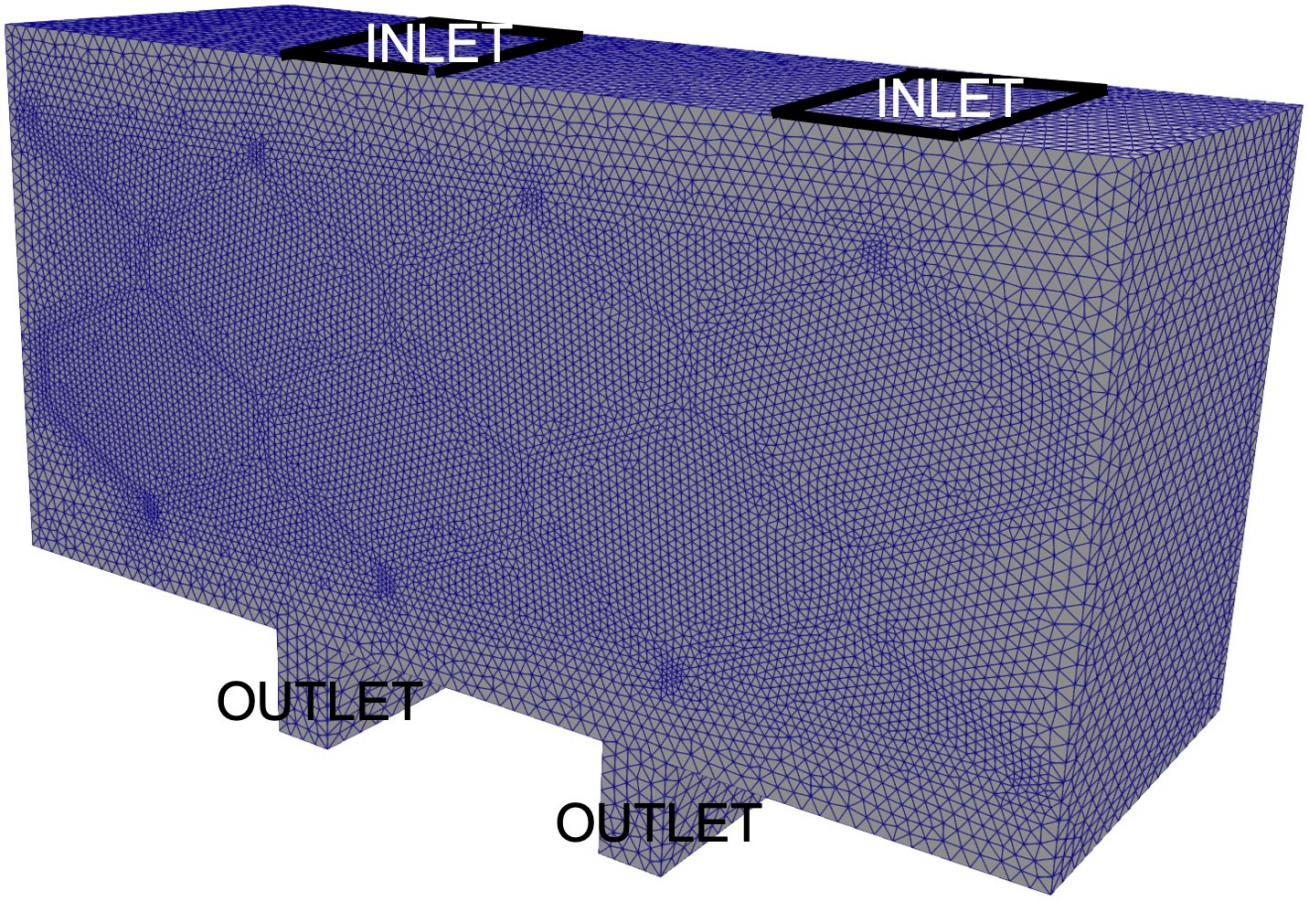
Computational Domain: The computational domain was discretized using conformal tetrahedral elements, resulting in a total of 926K elements.

We conclude this manuscript by demonstrating that the 2*D* results presented above accurately represent real-life 3*D* scenarios. In particular, we focus on a 3*D* scaffold corresponding to the hexagonal geometry considered in 2D, as shown in Fig 13 below. Each vertical slice of this geometry is identical to the 2*D* geometry shown at the bottom of Fig 4. In other words, the 3*D* geometry is obtained by extruding the 2*D* hexagonal geometry. The dimensions of the 3*D* spatial domain are 0.9 × 0.42 × 0.3 cm. Here 0.3 cm is the added thickness of the 3*D* domain. We maintained the same inlet and outlet setup as in the 2*D* case, and also kept all other parameters unchanged.

For the boundary conditions on the front and back wall of the computational domain we imposed the no-slip boundary condition for the free fluid velocity modeled by the time-dependent Stokes equations, and zero normal flux for Darcy velocity for the Biot equations. Displacement of the poroelastic matrix was also set to be equal to zero on the front and back walls of the chamber. On the rest of the boundary, we implemented the same boundary conditions as in the 2*D* case. Similarly, at the inlet, shown in Fig 13, Dirichlet boundary condition was imposed for the fluid velocity, and at the outlet, zero normal stress was imposed, as in the 2*D* case. The Dirichlet velocity imposed at the inlet is given by the uniform velocity profile with the magnitude of 5.25 cm/s, pointing downwards in the direction of the cell chamber. While this quantity is different from the one used in the 2*D* case, it is chosen so that the inflow flowrate is the same in the 2*D* case and the 3*D* case.

A monolithic solver reported in [13, 30, 31] was used to solve the 3*D* linearly coupled Stokes-Biot problem. Fig 13 depicts the computational domain, which was discretized using conformal tetrahedron elements. Taylor-Hood elements (P2-P1) were used for the free fluid velocity and pressure, P2 for the poroelastic structure displacement, P2 for Darcy velocity and P1 for Darcy pressure. We set the time step to be Δ*t* = 0.005 s, and allow the simulation to run until it reached a steady state at *T* = 1 s. As in the 2*D* case, the flow is driven by the pressure drop in the vertical direction generated by the inlet velocity and outlet normal stress data.

We examined the following quantities and compared them with the 2*D* simulation results: the free fluid velocity and pressure (at the inlet and outlet channels, and in the hexagonal geometry throughout the poroelastic medium), the Darcy velocity and Darcy pressure within the poroelastic structure, and the deformation of the poroelastic structure.

**Fig 14:**
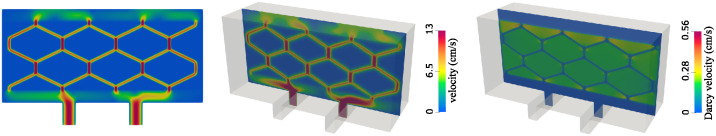
Velocity comparison. Left: 2*D* simulation showing free fluid and Darcy velocity ranging from 0 to 13 cm/s. Middle: 3*D* simulation showing velocity in the inlet, outlet and hexagonal geometry ranging from 0 to 13 cm/s. Right: 3*D* simulation showing only Darcy velocity ranging from 0 to 4.8 cm/s.

**Velocity**. Fig 14 shows a comparison of the velocity obtained using 2*D* simulations (left) and 3*D* simulations (middle and right). The middle panel shows only the free fluid (channel) velocity, while the right panel shows only Darcy velocity (poroelastic medium). Notice two different scales for the middle and right panel. We see that in both 2*D* and 3*D* scenarios the fluid velocity ranges from 0 to 13 cm/s, and that the highest velocity is achieved in the vertical channels, as expected due to the dominant pressure drop in the vertical direction.

**Fig 15:**
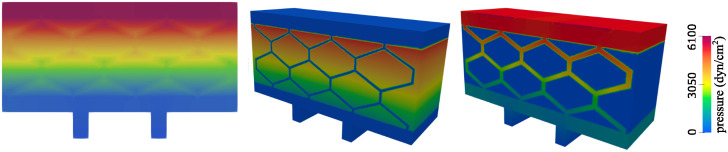
Pressure comparison. Left: 2*D* simulation showing free fluid and Darcy pressure. Middle: 3*D* simulation showing Darcy pressure. Right: 3*D* simulation showing pressure in the inlet, outlet and hexagonal geometry. The pressure scale is the same in all three panels—from 0 to around 6,000 dyn/cm^2^.

**Pressure**. Next we compared the fluid pressure. Fig 15 shows a comparison of the pressure obtained using 2*D* simulations (left) and 3*D* simulations (middle and right). In all three panels the pressure ranges from 0 to around 6,000 dyn/cm^2^. The panel on the left shows the combined 2*D* free fluid and Darcy pressure, while the figures on the right show separate 3*D* Darcy pressure (middle) and 3*D* free fluid pressure (right). Looking at both Stokes and Darcy regions, the pressure distributing obtained using 2*D* simulations is indeed, very close to the pressure distribution obtained using 3D simulations.

**Fig 16:**
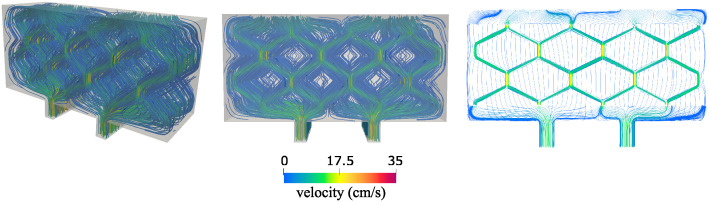
Streamlines generated by the fluid and Darcy velocity. The color of the streamlines corresponds to the velocity magnitude. Left: 3D simulations, side view. Middle: 3D simulations, front view. Right: 2D simulations.

**Streamlines**. To further compare 2*D* versus 3*D* spatial effects on the solution, we investigated the fluid velocity streamlines inside the entire 3*D* scaffold. This is shown in Fig 16. We can see that 2*D* effects are dominant over 3*D* effects, since the streamlines appear to be largely parallel to each other in the direction perpendicular to the plane containing the hexagonal geometry, indicating that 2*D* simulations approximate well the leading features of the fluid flow in the scaffold. The streamlines obtained using 2*D* simulations are shown in the right panel of Fig 16.

**Fig 17:**
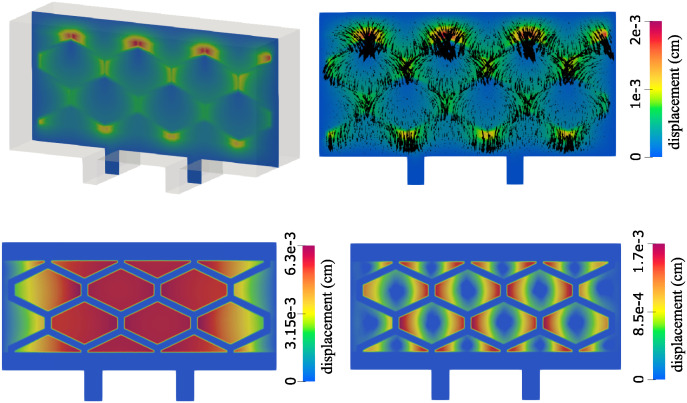
Displacement at the steady state. Top left: Magnitude of displacement obtained in 3*D*. Top right: Displacement vector field obtained in 3*D*. Bottom: The displacement obtained in 2*D* using a model with the spring term *γ*_*E*_***η*** (left) and by taking *γ*_*E*_ = 0 and fixing the center of each poroelastic region (right).

**Displacement**. Using 3*D* simulations we also investigated the total displacement of the poroelastic matrix as time increases toward the time at which the steady state solution is achieved. The magnitude of displacement at the steady state is shown in Fig 17 (top left) and the vectors showing the displacement vector field are shown in Fig 17 (top right). We observe that all cell compartments have expanded from their original shape, with the compartments closest to the inlet experiencing larger expansion than those closest to the outlet. We argue that this expansion is a result of flow saturation inside the cell compartments. This also results in a constriction of the original hexagonal flow channels, potentially increasing the proportion of flow passing through the cell compartments.

On the bottom of Fig 17 we also show the displacement obtained in 2*D*. The panel on the bottom left shows the displacement obtained using the structure model introduced in (3a), which has term *γ*_*E*_***η*** added. While this term damps the displacement magnitude, and help to keep the hexagonal and triangular structures from floating away, it does not resemble the displacement obtained in 3*D*. However, the magnitude of the displacement in both cases is comparable. On the bottom right panel we show the displacement obtained using an alternative approach, where we take *γ*_*E*_ = 0, but then fix the center of each interior poroelastic structure (displacement in the middle of each structure is equal to zero). This corresponds more closely to the 3*D* case, where the poroelastic structure is connected and fixed at the sides. In this case, we obtain a better resemblance to the 3*D* simulations. We note that neither of the 2*D* models are the same as the model used in 3*D*, which causes the results to differ. However, when the concentration obtained using either of these approaches is compared, no significant differences are found.

**Fig 18:**
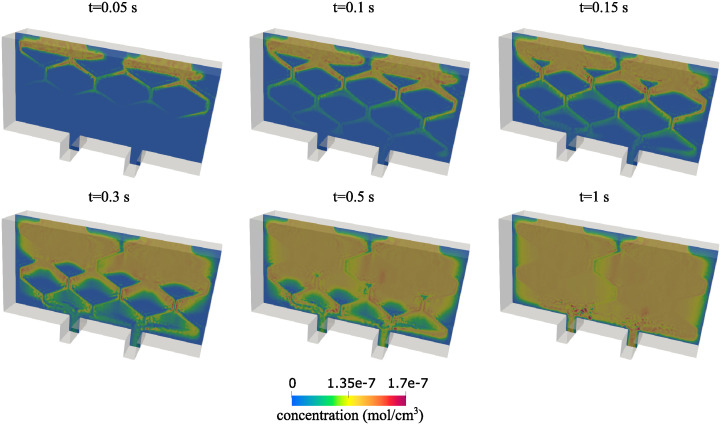
A series of screenshots depicting the transport of oxygen concentration from *t* = 0 s to *t* = 1 s, when the steady state solution is reached.

**Oxygen concentration**. Using 3*D* simulations we also investigated oxygen transport within the scaffold by solving the advection-reaction-diffusion problem described in Section 2.2. The advection-reaction-diffusion problem was solved with the time step of Δ*t* = 1*e* − 5*s*. In Fig 18 we illustrate the evolution of oxygen concentration from *t* = 0 to *t* = 0.15 seconds and then give the steady state solution of the oxygen concentration which is achieved at *t* = 1s. Fig 18 shows that indeed, the final oxygen concentration at *t* = 1 is nearly uniformly distributed throughout the chamber, except for the thin vertical region roughly half way between the two inlets. This is similar to the result obtained using 2*D* simulations, depicted in Fig 7 bottom right, which shows nearly uniform distribution of oxygen concentration except for a thin line located roughly in the middle between the two inlets. However, while the uninhibited maximal insulin production occurred in 97% of the total area in the 2*D* case, it only occurred in the 77% of total volume in the 3*D* case. This could be explained by the fact that due to the no flow conditions, the velocity and the concentration at the sides and the bottom of the domain are almost zero, and these regions account for a larger volume in 3*D* than in the 2*D* case. We conclude that, again, 2*D* simulations provide a good insight into oxygen concentration distribution within the cell chamber. Additionally, this result indicates that placing the two inlets closer to each other might improve uniform distribution of oxygen concentration within the chamber to include the central region between the two inlets.

## 7 Discussion: Conclusions

We devised a computational approach within the diffuse interface framework to explore the influence of scaffold architecture geometry on oxygen transport within biological scaffolds commonly employed in bioartificial organ engineering, with a specific focus on the bioartificial pancreas. To achieve this objective, we introduced a multi-physics model comprising a fluid flow component and an advection-reaction-diffusion model to analyze oxygen concentration within the scaffold. The fluid flow model incorporates the time-dependent Stokes equations coupled with the Biot equations, characterizing the behavior of a poroelastic medium representing the poroelastic hydrogel used in the scaffold design.

We explored three biologically inspired scaffold architecture geometries: vertically drilled channels, branching channels, and a hexagonal geometry. From a computational standpoint, one of the primary challenges in addressing problems with multiple varying geometries lies in generating new computational geometries and designing appropriate matrices for spatial discretization. These matrices describe the unknown variables, such as fluid velocity, pressure, and oxygen concentration, across different computational domains. To streamline mesh generation in complex geometries, we introduced a diffuse interface approach in this study. In the diffuse interface approach, the unknown variables are defined across the entire scaffold domain, with the specific geometry of the channel network captured by redefining only the phase-field function. This simplification proves crucial not only for our current work but also for future endeavors, where we aim to develop a geometric optimization solver. This solver will simplify the generation of numerous channel geometries to optimize scaffold architecture. It is important to notice that a drawback of the diffuse interface method is the large size of the discretization matrices since the number of unknowns at the discrete level is doubled. Furthermore, to obtain higher accuracy, the mesh is commonly refined around the interface, which leads to a higher number of degrees of freedom. However, in simulations where the permeability is small, a fine mesh around the interface is also needed when a sharp interface method is used to accurately resolve the pressure gradient between the Stokes and Biot regions. Such mesh must align with the interface if a sharp interface method is used, while this is not required for the diffuse interface method.

We demonstrated that the hexagonal geometry significantly outperforms both the branching channels’ network and the classical vertical channel geometries. Our analysis indicates that the superior performance of the hexagonal geometry stems from the relatively large angle between the dominant channel flow direction and the channel-hydrogel interface. This configuration results in a larger Darcy velocity, thereby facilitating enhanced advection-mediated oxygen supply to the transplanted cells. This study is significant because recent developments in hydrogel fabrication make it now possible to control hydrogel rheology [2, 5], utilizing the computational results to generate optimized scaffold architectures.

Our future work includes the design of a geometric optimization algorithm for optimal scaffold architecture design. In case of geometric optimization, or just a moving domain problem, we expect it to be necessary to adapt the mesh when the phase function changes significantly. This can be done at each step, or every few steps, depending on how fast the phase field function evolves, by using the gradient of Φ.
